# Combined pulmonary fibrosis and emphysema: an increasingly recognized condition* **

**DOI:** 10.1590/S1806-37132014000300014

**Published:** 2014

**Authors:** Olívia Meira Dias, Bruno Guedes Baldi, André Nathan Costa, Carlos Roberto Ribeiro Carvalho

**Affiliations:** Department of Cardiorespiratory Diseases, Instituto do Coração - InCor, Heart Institute - University of São Paulo School of Medicine Hospital das Clínicas, São Paulo, Brazil; Department of Cardiorespiratory Diseases, Instituto do Coração - InCor, Heart Institute - University of São Paulo School of Medicine Hospital das Clínicas, São Paulo, Brazil; Department of Cardiorespiratory Diseases, Instituto do Coração - InCor, Heart Institute - University of São Paulo School of Medicine Hospital das Clínicas, São Paulo, Brazil; Department of Cardiorespiratory Diseases, Instituto do Coração - InCor, Heart Institute - University of São Paulo School of Medicine Hospital das Clínicas, São Paulo, Brazil

**Keywords:** Pulmonary fibrosis, Emphysema, Hypertension, pulmonary, Lung diseases, interstitial

## Abstract

Combined pulmonary fibrosis and emphysema (CPFE) has been increasingly recognized in the literature. Patients with CPFE are usually heavy smokers or former smokers with concomitant lower lobe fibrosis and upper lobe emphysema on chest HRCT scans. They commonly present with severe breathlessness and low DLCO, despite spirometry showing relatively preserved lung volumes. Moderate to severe pulmonary arterial hypertension is common in such patients, who are also at an increased risk of developing lung cancer. Unfortunately, there is currently no effective treatment for CPFE. In this review, we discuss the current knowledge of the pathogenesis, clinical characteristics, and prognostic factors of CPFE. Given that most of the published data on CPFE are based on retrospective analysis, more studies are needed in order to address the role of emphysema and its subtypes; the progression of fibrosis/emphysema and its correlation with inflammation; treatment options; and prognosis.

## Introduction

Idiopathic pulmonary fibrosis (IPF) and pulmonary emphysema are distinct clinicopathological entities that pulmonologists have long been familiar with. Since the advent of HRCT, the combination of these two conditions has been increasingly described and has been proven to be a prevalent and distinct entity rather than a rare coincidence. 

The association of IPF and emphysema was initially described in 1990 by Wiggins et al.,^(^ ^1^ ^)^ who described eight heavy smokers with fibrosis and upper lobe emphysema on HRCT scans, together with severe breathlessness, strikingly low DLCO, and preserved lung volumes. In 2005, Grubstein et al.^(^ ^2^ ^)^ reported an association of fibrosis with emphysema in eight patients, their clinical and functional findings being similar to those of the aforementioned study. The authors also found moderate to severe pulmonary arterial hypertension (PAH) and postulated that smoking is a factor linking emphysema, pulmonary fibrosis, and pulmonary vascular disease.^(^ ^2^ ^)^ The term combined pulmonary fibrosis and emphysema (CPFE) was first used in 2005 by Cottin et al.,^(^ ^3^ ^)^ who characterized a homogeneous group of 61 patients with CT findings of emphysema in the upper zones and interstitial lung disease (ILD) with pulmonary fibrosis in the lower lobes.

When CPFE was first described, patients with other ILDs were excluded from the study.^(^ ^3^ ^)^ Later on, CPFE was described in patients with other ILDs, such as connective tissue disease (CTD)-associated ILD,^(^ ^4^ ^-^ ^7^ ^)^ as well as in patients with microscopic polyangiitis.^(^ ^8^ ^)^

Studies have shown that patients with CPFE associated with CTDs (especially rheumatoid arthritis and systemic sclerosis) are significantly younger than their idiopathic CPFE counterparts, are predominantly female, and have less DLCO impairment.^(^ ^4^ ^)^ One group of authors found elevated serum antinuclear antibodies with or without positive perinuclear antineutrophil cytoplasmic antibodies in CPFE patients when compared with IPF patients without emphysema, those with positive autoimmune markers exhibiting greater infiltration of CD20+ B cells forming lymphoid follicles in fibrotic lung tissue and improved survival when compared with those with negative autoimmune markers.^(^ ^9^ ^)^

Given that tobacco exposure seems to modulate an underlying inflammatory response in patients with ILD, CPFE should be categorized as a pattern associated with other pulmonary diseases rather than as a primary idiopathic syndrome, a classification similar to the usual interstitial pneumonia (UIP) pattern in other fibrotic ILDs. In other words, the recognition of a CPFE pattern should also prompt the investigation of secondary autoimmune diseases and CTDs.

Patients with CPFE are predominantly male, with a history of heavy tobacco exposure, and usually present with severe breathlessness and cough. Physical examination reveals "Velcro" crackles at the lung bases and digital clubbing.^(^ ^3^ ^,^ ^10^ ^)^ Pulmonary hypertension is a hallmark of the syndrome and determines poor prognosis.^(^ ^10^ ^)^ Between January of 2006 and December of 2013, 17 patients were diagnosed with CPFE at our interstitial lung disease outpatient clinic, and the data are summarized in Table 1. In accordance with the literature, our patients were predominantly male (88%), the mean age at diagnosis being 68 years. All of the patients presented with tobacco exposure and dyspnea at diagnosis. Almost half of the patients had pulmonary hypertension diagnosed by echocardiography. Few (6%) had a diagnosis of lung cancer, and 12% died during the follow-up period.

**Table 1:** - Characteristics of 17 patients with combined pulmonary fibrosis and emphysema treated at the Interstitial Lung Disease Outpatient Clinic of the University of São Paulo School of Medicine Hospital das Clínicas between 2006 and 2013, together with the clinical manifestations of the disease.a

Characteristic	Result
Male/Female	15 (88)/2 (12)
Age at diagnosis, yearsb	68 ± 7
Tobacco exposure	17 (100)
Dyspnea at diagnosis	17 (100)
Pulmonary hypertension at diagnosis	8 (47)
Hypoxemia at diagnosis	11 (65)
Lung cancer during follow-up	1 (6)
Deaths during follow-up	2 (12)

a Values expressed as n (%), except where otherwise indicated.

b Values expressed as mean ± SD.

### Pathogenesis

The pathogenesis of CPFE has yet to be elucidated. Tobacco exposure per se can be an important fibrogenic stimulus, smoking having been shown to play a key role in the pathogenesis of several ILDs, including respiratory bronchiolitis-associated ILD (RB-ILD), desquamative interstitial pneumonia, pulmonary Langerhans cell histiocytosis, and, possibly, IPF. 

Washko et al. conducted a lung cancer screening study involving a large cohort of COPD patients and found interstitial lung abnormalities on HRCT scans in up to 8% of smokers.^(^ ^11^ ^)^ Likewise, Katzenstein et al. reported frequent and severe interstitial fibrosis in over half of lobectomy specimens excised for lung cancer from smokers with no clinical evidence of ILD, even in those patients in whom emphysema was the only CT finding.^(^ ^12^ ^)^

Those histological findings characterized a distinct, non-classifiable ILD, which Katzenstein et al. designated "smoking related-interstitial fibrosis", characterized by thickening of alveolar septa by fibrosis composed mostly of hyalinized eosinophilic collagen bundles and surrounding enlarged airspaces of emphysema, as well as by signs of respiratory bronchiolitis.^(^ ^12^ ^)^ Although follow-up was short, the clinical progression seemed to be particularly different from that of IPF, with indolent fibrosis and better survival rates, reinforcing the idea of a different disease.^(^ ^12^ ^)^

It is reasonable to assume that the lung parenchyma shows different patterns of injury and repair in response to tobacco exposure. The different phenotypes of lesions secondary to tobacco exposure depend on the balance of apoptosis, proteolysis, and fibrosis. Patients in whom genes related to connective tissue synthesis, structural constituents of the cytoskeleton, and cell adhesion are overexpressed typically display a fibrogenic phenotype, such as that found in patients with UIP; however, a different inflammatory response to smoking-associated cellular damage (destruction and repair of cells, vessels, and pneumocytes) leads to destruction of lung parenchyma, culminating in pulmonary emphysema.^(^ ^13^ ^)^ A combination of these two patterns of response can be found in patients with CPFE and has recently been demonstrated by gene expression analysis of fibrotic and emphysematous lesions in such patients.^(^ ^13^ ^)^

The role of environmental exposure as a potential trigger of lung injury is also plausible, given that some CPFE patients have had significant exposure to agrochemical compounds that cause airway damage and ILD in genetically susceptible smokers.^(^ ^14^ ^)^ Some authors have described CPFE as an occupational disease, e.g., in patients exposed to talc^(^ ^15^ ^)^ and in welders.^(^ ^16^ ^)^

The signaling pathways to these responses are unknown. Laboratory animal studies have demonstrated that oxidative stress inducing inflammatory cell activation, elevated matrix metalloproteinase levels causing proteolytic activity,^(^ ^17^ ^,^ ^18^ ^)^ and overexpression of other mediators, such as PDGF,^(^ ^19^ ^)^ TNF-α, and TGF-β,^(^ ^20^ ^,^ ^21^ ^)^ are potential pathways explaining the lesions that lead to emphysema and fibrosis. A study analyzing inflammatory mediators in BAL fluid from patients with IPF showed significantly higher concentrations of chemokine (C-X-C motif) ligand 5 and chemokine (C-X-C motif) ligand 8 in those with concomitant HRCT findings of emphysema.^(^ ^22^ ^)^ These chemokines are associated with neutrophil accumulation in airspaces and suggest a different pathway of inflammation leading to the development of emphysematous changes superimposed on pulmonary fibrosis.^(^ ^22^ ^)^

Genetic mutations have been described in CPFE patients with and without a significant smoking history.^(^ ^6^ ^,^ ^23^ ^)^ This indicates that risk factors other than tobacco smoking are associated with the development of CPFE or that tobacco smoking can be a triggering factor in susceptible patients. Plausible genetic pathways have been confirmed in case reports in which mutations in the surfactant protein C gene were identified in a 32-year-old female who had never smoked^(^ ^23^ ^)^ and an ABCA3 mutation was identified in a 41-year-old male nonsmoker,^(^ ^24^ ^)^ with CT findings identical to those in CPFE patients. These mutations are known to cause dysfunction of surfactant homeostasis and, consequently, injury or death of alveolar epithelial type II cells and myofibroblast proliferation.^(^ ^23^ ^)^ Finally, reports have described CPFE features in a family with inherited telomerase mutations.^(^ ^25^ ^)^

These findings reinforce the idea of a combination of genetic predisposition and a triggering exposure (smoking) in susceptible individuals leading to continued damage to alveolar epithelial cells that cannot be properly repaired, initiating a vicious cycle of attempts at alveolar regeneration and uncontrolled activation of fibrosis proliferation and parenchymal destruction.^(^ ^26^ ^)^

### Imaging studies in CPFE

Imaging studies are essential for the diagnosis of CPFE. Although routine chest X-rays are not as sensitive as HRCT scans, they can reveal an interstitial pattern predominantly in the subpleural and basal lung regions, with hyperlucency in the lung apices corresponding to emphysematous areas.

**Figure 1:**
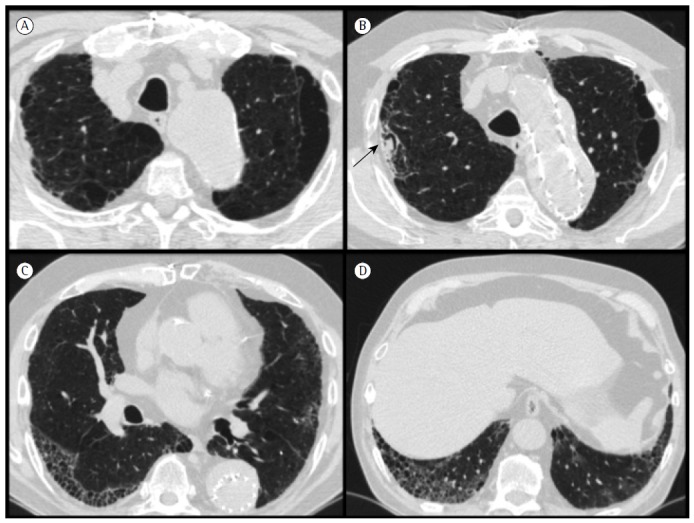
CT scan of the chest of a 67-year-old female patient with combined pulmonary fibrosis and emphysema, showing centrilobular and paraseptal emphysema in the upper lobes (A and B), as well as ground-glass opacities, traction bronchiectasis, and honeycombing in the lower lobes (C and D). Note an aspergilloma in one of the paraseptal bullae in the right upper lobe (black arrow, in B)

**Figure 2:**
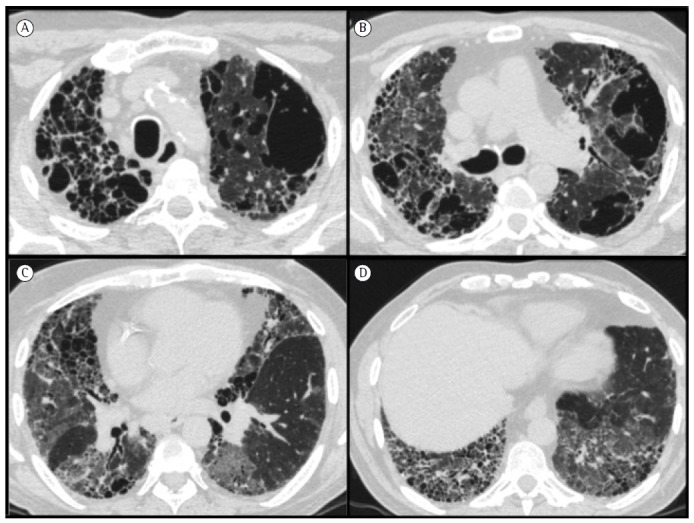
CT scan of the chest of a 70-year-old male patient with combined pulmonary fibrosis and emphysema and acute exacerbation of interstitial disease. Note predominantly paraseptal emphysema in the lung apices, with architectural destruction of the lung parenchyma (A and B). Extensive areas of ground-glass opacity and honeycombing can be seen in the lower lobes (C and D)

The mainstay of the diagnosis of CPFE, HRCT scans typically show centrilobular or paraseptal emphysema in the upper lobes, as well as reticular opacities, traction bronchiectasis, septal thickening, ground-glass opacities, and honeycombing in the lower lobes(3) (Figures 1 and 2). Although UIP is the most common CT pattern, some patients have ground-glass opacities that are more extensive than expected for a UIP pattern and are therefore suggestive of nonspecific interstitial pneumonia, RB-ILD, and even desquamative interstitial pneumonia.^(^ ^3^ ^)^

Brillet et al.^(^ ^27^ ^)^ described patterns of distribution of fibrosis and emphysema in patients with CPFE other than those initially described: a progressive transition from apical emphysema to a zone of transition between bullae and honeycombing; paraseptal emphysema with areas of fibrosis; and separate processes with independent areas of fibrosis and emphysema.^(^ ^27^ ^)^

### Lung function in CPFE

Regarding the lung function of patients with CPFE, spirometry can be normal or show mild abnormalities, FVC, FEV_1_, and TLC values usually being within normal ranges or slightly altered.^(^ ^3^ ^,^ ^28^ ^)^ The FEV_1_/FVC ratio can be within or slightly below the normal range. Severely impaired DLCO and hypoxemia during exercise are common.^(^ ^3^ ^,^ ^28^ ^)^

Silva et al. reported similar findings in a retrospective cohort of 11 Brazilian patients, spirometry having revealed normal lung volumes in 7.^(^ ^29^ ^)^ At our facility, 17 CPFE patients were evaluated, and spirometry showed normal lung function and lung volumes in 12%, an obstructive pattern in 18%, and a restrictive pattern in 47%. All patients had reduced DLCO (Table 2).

**Table 2:** - Pulmonary function test results at diagnosis in 17 patients with combined pulmonary fibrosis and emphysema treated at the Interstitial Lung Disease Outpatient Clinic of the University of São Paulo Hospital das Clínicas between 2006 and 2013.a

Variable	Result
Obstructive pattern	3 (18)
Restrictive pattern	8 (47)
Air trapping	4 (24)
Normal spirometry and pulmonary volumes	2 (12)
Reduced DLCO	17 (100)
FVC, % predictedb	79 ± 16
FEV1, % predictedb	79 ± 14
FEV1/FVCb	0.77 ± 0.08
TLC, % predictedb	73 ± 17
RV/TLCb	0.36 ± 0.06
DLCO, % predictedb	30 ± 14

a Values expressed as n (%), except where otherwise indicated.

b Values expressed as mean ± SD.

In a five-year follow-up study of 16 CPFE patients, the annual decline in FVC, DLCO, and DLCO/alveolar volume was significantly higher than was that found in a group of COPD patients. ^(^ ^30^ ^)^ In another study, the rate of decline in lung volume was found to be considerably lower in patients with CPFE than in those with IPF.^(^ ^31^ ^)^ Although DLCO values were lower in the patients with CPFE than in those with IPF, the annual rate of decline in DLCO was also significantly lower in the former. There were no differences between the two groups in terms of survival.^(^ ^31^ ^)^

One possible explanation for normal or subnormal spirometry results despite severe impairment in DLCO is that hyperinflation and greater lung compliance as a result of loss of elasticity in the areas of emphysema can compensate for the losses in volume and lung compliance caused by fibrosis. Another plausible explanation is that fibrosis prevents the early small airway closure observed in patients with emphysema.

Although a single spirometry test can underestimate the severity of the disease, Schmidt et al. demonstrated that a progressive approach, with a longitudinal decline in FEV_1_, can accurately define disease progression and predict mortality in CPFE patients.^(^ ^32^ ^)^ In addition, the authors found a correlation between the extent of emphysema on HRCT scans and the decline in FEV_1_.^(^ ^32^ ^)^

### PAH in CPFE

The prevalence of PAH is exceedingly high in patients with CPFE, and PAH correlates with worse survival.^(^ ^3^ ^,^ ^33^ ^,^ ^34^ ^)^ The prevalence of PAH in CPFE patients varies from 47% to 90%, being considerably higher than that in patients with COPD or IPF alone.^(^ ^18^ ^)^ Indeed, the five-year survival rate in a study involving CPFE patients was 25% in those with PAH (as measured by transthoracic echocardiography), being 75% in those without PAH.^(^ ^28^ ^)^ In another study, the finding of severe PAH on echocardiography was associated with an increased risk of death.^(^ ^35^ ^)^

Mejia et al. conducted a study in Mexico, in which PAH was assessed by transthoracic echocardiography in a cohort of patients with IPF (with and without emphysema). Not only was PAH more prevalent in the patients with CPFE, but it was also responsible for a worse prognosis. Another important finding was that the amount of emphysema on CT scans was directly correlated with a higher estimated systolic pulmonary artery pressure.^(^ ^34^ ^)^

Although transthoracic echocardiography is an operator-dependent imaging modality and lacks accuracy in the diagnosis of PAH in patients with advanced lung disease, including COPD and IPF, it seems to have a good correlation with right heart catheterization studies in CPFE patients, being therefore an effective screening tool for PAH in such patients.^(^ ^18^ ^,^ ^34^ ^)^

Cottin et al. retrospectively characterized PAH by means of right heart catheterization in 40 patients with CPFE. Higher pulmonary vascular resistance, higher HR, lower cardiac index, and lower DLCO were associated with a worse prognosis, the one-year survival rate being 60%. Although an evaluation of the effect of treatment was not the primary objective of the study, none of the available treatments improved survival.^(^ ^33^ ^)^

Novel noninvasive methods for the diagnosis and quantification of PAH in CPFE patients have been proposed, including time-resolved magnetic resonance angiography,^(^ ^35^ ^)^ which allows anatomic imaging of the pulmonary vasculature and evaluation of hemodynamic parameters.^(^ ^35^ ^)^ Using this technique, Sergiacomi et al. prospectively studied 18 CPFE patients using pulmonary arterial mean transit time and time to peak enhancement as surrogate parameters for hemodynamic data (mean pulmonary artery pressure and pulmonary vascular resistance), which were obtained through right heart catheterization performed three days before time-resolved magnetic resonance angiography was performed.^(^ ^35^ ^)^ Pulmonary arterial mean transit time and time to peak enhancement showed good correlation with the invasive parameters.^(^ ^35^ ^)^

### Treatment and prognosis

As is the case with IPF, there is currently no effective treatment for CPFE, with the exception of smoking cessation and lung transplantation (for patients with advanced disease). Bronchodilators, however, can be prescribed to patients with a positive response to bronchodilators in pulmonary function tests. Cottin et al.^(^ ^4^ ^)^ recommended the use of N-acetylcysteine (1.8 g/day) on the basis of the results of studies investigating IPF. Oral corticosteroids and immunosuppressants have been considered an option in the setting of CTD-associated CPFE; however, no randomized trials have been conducted.^(^ ^4^ ^)^ Lung transplantation is the only option that can improve survival.

The major causes of death in patients with CPFE are chronic respiratory failure, PAH,^(^ ^25^ ^,^ ^26^ ^)^ acute exacerbation,^(^ ^3^ ^,^ ^4^ ^)^ and lung cancer.^(^ ^36^ ^,^ ^37^ ^)^ Usui et al. found an 8.9% prevalence of CPFE in 1,143 consecutive patients with primary lung cancer in Japan.^(^ ^36^ ^)^ The authors showed that, in comparison with patients with fibrosis or emphysema alone, CPFE patients had a worse survival and an increased incidence of acute exacerbation after surgery.^(^ ^36^ ^)^

Variations in DLCO, hypoxemia, digital clubbing,^(^ ^10^ ^)^ mean pulmonary artery pressure,^(^ ^26^ ^,^ ^27^ ^)^ and decline in FEV_1_^(^ ^32^ ^)^ are considered better surrogates for disease progression and higher risk of mortality, allowing earlier evaluation for transplantation. Recently, Chiba et al. demonstrated that two biomarkers of fibrosis, namely KL-6 and surfactant protein D, are good indicators of the extent of fibrosis in patients with CPFE.^(^ ^38^ ^)^ High KL-6 and surfactant protein D levels were found to correlate negatively with all lung volumes and with DLCO.^(^ ^38^ ^)^ Kishaba et al. demonstrated that high levels of KL-6 are predictors of acute exacerbations in patients with CPFE.^(^ ^10^ ^)^

The prognosis and overall mortality of CPFE patients in comparison with those of IPF and COPD patients is still a matter of debate. Different enrollment criteria, duration of follow-up, heterogeneity of patients (including type of emphysema), retrospective data analysis, and lead-time bias might explain the heterogeneity of results.

Todd et al.^(^ ^39^ ^)^ and Todd & Atamas^(^ ^40^ ^)^ examined the extent and type of emphysema in a subset of patients with pulmonary fibrosis and found that patients with a combination of fibrosis with centrilobular or mixed (centrilobular and paraseptal) emphysema had better survival rates than did those with pulmonary fibrosis without emphysema, those with trivial emphysema, and those with advanced paraseptal emphysema.^(^ ^39^ ^,^ ^40^ ^)^ The pattern of emphysema and its extent seem to correlate with disease severity. The reasons for these findings remain unknown. One possible explanation is that patients with CPFE tend to have somewhat preserved lung volumes compared with patients with fibrosis alone; however, no retrospective studies found any correlation between preserved lung volumes (as determined by pulmonary function tests) and better survival in patients with CPFE. In addition, there were no differences in DLCO among those groups of patients.^(^ ^39^ ^,^ ^40^ ^)^

Another hypothesis for centrilobular emphysema acting as a "protective factor" is based on the fact that centrilobular emphysema is essentially caused by tobacco exposure. Some in vitro studies have shown that the proinflammatory cytokines seen in cases of cigarette smoking and emphysema have antifibrotic properties; therefore, such patients might have smaller areas of fibrosis and a better prognosis.^(^ ^19^ ^)^ Paraseptal emphysema might represent another lung response to smoking, leading to severe pulmonary fibrosis, or simply reflect a greater extent of fibrosis in lower lung zones, exerting traction on lung tissue located in the apices. However, the fibrotic areas seen on chest X-rays do not precede the onset of emphysema, indicating that both processes probably occur simultaneously. 

Corroborating this theory, Kurashima et al. found that a greater extent of emphysema on CT scans correlated with better pulmonary function parameters and a better prognosis in comparison with those in a group of patients with IPF.^(^ ^41^ ^)^ The authors did not classify emphysema into subtypes and suggested that emphysema is a protective factor in patients with CPFE.^(^ ^41^ ^)^ Similar findings were reported by Ando et al.^(^ ^42^ ^)^ in a study examining the relationship between pulmonary function and CT quantification of emphysema and fibrosis in CPFE, the authors having concluded that pulmonary fibrotic changes contribute more to the progression of CPFE than does emphysema.^(^ ^42^ ^)^

The mortality rates for CPFE and IPF are similar. ^(^ ^43^ ^,^ ^44^ ^)^ Recently, Ryerson et al. compared CPFE-IPF patients with non-CPFE IPF patients.^(^ ^45^ ^)^ Although the patients with CPFE had a more extensive smoking history, greater oxygen requirements, higher pulmonary artery pressure, less restrictive physiology, and lower diffusing capacity, there was no significant difference in mortality between the two groups.^(^ ^45^ ^)^

### Final considerations

Pulmonologists have been accustomed to recognizing fibrosis and emphysema as two well-defined diseases. However, a large body of evidence has shown that an overlap can exist, CPFE being therefore a new entity, with unique features. In view of the fact that most studies investigating CPFE have had a retrospective design, more studies are needed to address the role of emphysema and its subtypes, the progression of fibrosis/emphysema and its correlation with inflammation, treatment options (including the treatment of PAH), and prognosis in CPFE patients. A deeper understanding of the pathophysiology and progression of CPFE is urgently required in order to improve its management, given that there is currently no effective treatment for the disease.
