# Supplementary material for: Combined pulmonary fibrosis and emphysema: an increasingly recognized condition
Source: J Bras Pneumol. 2014 May-Jun;40(3):304–12. doi: 10.1590/S1806-37132014000300014 (PMC4109203; doi:10.1590/S1806-37132014000300014)
Supplement: Translation

# Translation: Combinação de fibrose pulmonar e enfisema: uma doença cada vez mais reconhecida*

**Authors:** Olívia Meira Dias, Bruno Guedes Baldi, André Nathan Costa, Carlos Roberto Ribeiro Carvalho

**Affiliations:** Departamento de Cardiopneumologia, Instituto do Coração - InCor - Hospital das Clínicas, Faculdade de Medicina da Universidade de São Paulo, São Paulo (SP) Brasil; Departamento de Cardiopneumologia, Instituto do Coração - InCor - Hospital das Clínicas, Faculdade de Medicina da Universidade de São Paulo, São Paulo (SP) Brasil; Departamento de Cardiopneumologia, Instituto do Coração - InCor - Hospital das Clínicas, Faculdade de Medicina da Universidade de São Paulo, São Paulo (SP) Brasil; Departamento de Cardiopneumologia, Instituto do Coração - InCor - Hospital das Clínicas, Faculdade de Medicina da Universidade de São Paulo, São Paulo (SP) Brasil

**Keywords:** Fibrose pulmonar, Enfisema, Hipertensão pulmonar, Doenças pulmonares intersticiais

## Abstract

A combinação de fibrose pulmonar e enfisema (CFPE) é cada vez mais reconhecida na literatura. Os pacientes são geralmente fumantes pesados ou ex-fumantes nos quais a TCAR de tórax revela enfisema nos lobos superiores e, concomitantemente, fibrose nos lobos inferiores. Esses pacientes comumente apresentam dispneia grave e baixa DLCO, não obstante os volumes pulmonares relativamente preservados em exames espirométricos. Hipertensão arterial pulmonar de moderada a grave e aumento da incidência de câncer de pulmão também são comuns nesses pacientes. Infelizmente, ainda não existe um tratamento eficaz para a CFPE. O objetivo desta revisão é discutir o que se sabe atualmente a respeito da patogênese, das características clínicas e dos fatores prognósticos da CFPE. Como a maioria dos dados publicados baseia-se em análise retrospectiva, são necessários mais estudos sobre o papel do enfisema e seus subtipos, a progressão da fibrose/enfisema e sua correlação com a inflamação, as opções de tratamento e o prognóstico em pacientes com CFPE.

## Introdução

A fibrose pulmonar idiopática (FPI) e o enfisema pulmonar são entidades clínico-patológicas distintas com as quais os pneumologistas há muito estão familiarizados. Desde o advento da TCAR, a combinação dessas duas doenças é cada vez mais descrita e provou ser uma entidade distinta e prevalente, e não uma rara coincidência. 

A associação de FPI e enfisema foi inicialmente descrita em 1990 por Wiggins et al.,^(^ ^1^ ^)^ que descreveram oito fumantes pesados com fibrose e enfisema nos lobos superiores na TCAR, além de falta de ar grave, DLCO surpreendentemente baixa e volumes pulmonares preservados. Em 2005, Grubstein et al.^(^ ^2^ ^)^ relataram uma associação de fibrose e enfisema em oito pacientes, com achados clínicos e funcionais semelhantes aos do estudo supracitado. Os autores observaram também hipertensão arterial pulmonar (HAP) de moderada a grave e propuseram que o tabagismo é um elo entre hipertensão e enfisema, fibrose pulmonar e doença vascular pulmonar.^(^ ^2^ ^)^ O termo combinação de fibrose pulmonar e enfisema (CFPE) foi usado pela primeira vez em 2005 por Cottin et al.,^(^ ^3^ ^)^ que caracterizaram um grupo homogêneo de 61 pacientes com achados tomográficos de enfisema nas zonas superiores e doença pulmonar intersticial (DPI) com fibrose pulmonar nos lobos inferiores.

Quando a CFPE foi descrita pela primeira vez, pacientes com outras DPI foram excluídos do estudo.^(^ ^3^ ^)^ Mais tarde, a CFPE foi descrita em pacientes com outras DPI, tais como a DPI relacionada com doença do tecido conjuntivo (DTC),^(^ ^4^ ^-^ ^7^ ^)^ bem como em pacientes com poliangiite microscópica.^(^ ^8^ ^)^

Estudos mostram que pacientes com CFPE relacionada com DTC (especialmente artrite reumatoide e esclerose sistêmica) são significativamente mais jovens que os com CFPE idiopática, são na maioria das vezes do sexo feminino e apresentam menos perda da DLCO.^(^ ^4^ ^)^ Um grupo de autores encontrou anticorpos antinucleares séricos elevados com ou sem positividade para anticorpos anticitoplasma de neutrófilos com padrão perinuclear em pacientes com CFPE ao compará-los com pacientes com FPI sem enfisema, sendo que aqueles com marcadores autoimunes positivos apresentaram maior infiltração de células CD20+ B formando folículos linfoides no tecido pulmonar fibrótico e melhor sobrevida em comparação com aqueles com marcadores autoimunes negativos.^(^ ^9^ ^)^

Como a exposição ao tabaco parece modular uma resposta inflamatória subjacente em pacientes com DPI, a CFPE deveria ser considerada um padrão relacionado com outras doenças pulmonares, e não uma síndrome idiopática primária, uma classificação semelhante ao padrão de pneumonia intersticial usual (PIU) em outras DPI fibróticas. Em outras palavras, o reconhecimento de um padrão de CFPE também deveria levar à investigação de doenças autoimunes secundárias e DTC. 

Pacientes com CFPE são na maioria das vezes do sexo masculino, com história de exposição intensa ao tabaco, e geralmente apresentam falta de ar grave e tosse. O exame físico revela crepitações do tipo "velcro" nas bases pulmonares e baqueteamento digital.^(^ ^3^ ^,^ ^10^ ^)^ A hipertensão pulmonar é um atributo marcante da síndrome e determina um prognóstico ruim.^(^ ^10^ ^)^ Entre janeiro de 2006 e dezembro de 2013, 17 pacientes receberam diagnóstico de CFPE em nosso ambulatório de doenças pulmonares intersticiais, e os dados estão resumidos na Tabela 1. Conforme a literatura, a maioria de nossos pacientes era do sexo masculino (88%), com média de idade de 68 anos no momento do diagnóstico. Todos os pacientes apresentaram exposição ao tabaco e dispneia no momento do diagnóstico. Quase metade dos pacientes apresentou hipertensão pulmonar diagnosticada por ecocardiografia. Poucos (6%) receberam diagnóstico de câncer de pulmão, e 12% morreram durante o período de acompanhamento.

**Tabela 1:** Características de 17 pacientes com combinação de fibrose pulmonar e enfisema tratados no Ambulatório de Doenças Pulmonares Intersticiais do Hospital das Clínicas da Faculdade de Medicina da Universidade de São Paulo entre 2006 e 2013, bem como as manifestações clínicas da doença.a

Característica	Resultado
Masculino/Feminino	15 (88)/2 (12)
Idade no momento do diagnóstico, anosb	68 ± 7
Exposição ao tabaco	17 (100)
Dispneia no momento do diagnóstico	17 (100)
Hipertensão pulmonar no momento do diagnóstico	8 (47)
Hipoxemia no momento do diagnóstico	11 (65)
Câncer de pulmão durante o acompanhamento	1 (6)
Óbitos no momento durante o acompanhamento	2 (12)

a Valores expressos em n (%), exceto onde indicado

b Valores expressos em média ± dp.

### Patogênese

A patogênese da CFPE ainda não foi elucidada. A exposição ao tabaco, por si só, pode ser um importante estímulo fibrogênico; demonstrou-se que o tabagismo desempenha um papel fundamental na patogênese de diversas DPI, tais como a DPI relacionada com bronquiolite respiratória (DPI-BR), a pneumonia intersticial descamativa, a histiocitose pulmonar de células de Langerhans e, possivelmente, a FPI. 

Washko et al. realizaram um estudo de rastreamento de câncer de pulmão em uma grande coorte de pacientes com DPOC e encontraram anormalidades pulmonares intersticiais na TCAR de até 8% dos fumantes.^(^ ^11^ ^)^ Da mesma forma, Katzenstein et al. relataram fibrose intersticial grave e frequente em mais da metade dos espécimes de lobectomia ressecados por câncer de pulmão em fumantes sem evidência clínica de DPI, mesmo naqueles pacientes nos quais o único achado tomográfico era o enfisema.^(^ ^12^ ^)^

Esses achados histológicos caracterizaram uma DPI distinta, não classificável, que Katzenstein et al. denominaram "fibrose intersticial relacionada com o tabagismo", caracterizada por espessamento de septos alveolares por fibrose composta principalmente por feixes de fibras colágenas eosinofílicas hialinas e envolvendo espaços aéreos aumentados de enfisema, bem como por sinais de bronquiolite respiratória.^(^ ^12^ ^)^ Embora o acompanhamento tenha sido curto, a evolução clínica pareceu ser particularmente diferente daquela da FPI, com fibrose indolente e melhores taxas de sobrevida, reforçando a ideia de uma doença diferente.^(^ ^12^ ^)^

É razoável supor que o parênquima pulmonar apresente diferentes padrões de lesão e reparação em resposta à exposição ao tabaco. Os diferentes fenótipos de lesões secundárias à exposição ao tabaco dependem do equilíbrio entre apoptose, proteólise e fibrose. Os pacientes nos quais genes relacionados com síntese de tecido conjuntivo, componentes estruturais do citoesqueleto e adesão celular estão superexpressos tipicamente exibem um fenótipo fibrogênico, como o encontrado em pacientes com PIU; no entanto, uma resposta inflamatória diferente ao dano celular relacionado com o tabagismo (destruição e reparação de células, vasos e pneumócitos) leva à destruição do parênquima pulmonar, culminando com enfisema pulmonar.^(^ ^13^ ^)^ Uma combinação desses dois padrões de resposta pode ser encontrada em pacientes com CFPE e foi recentemente demonstrada por meio de análise de expressão gênica de lesões fibróticas e enfisematosas em tais pacientes.^(^ ^13^ ^)^

O papel da exposição ambiental como potencial gatilho de lesão pulmonar também é plausível, já que alguns pacientes com CFPE apresentam significativa exposição a compostos agroquímicos que causam danos às vias aéreas e DPI em fumantes geneticamente suscetíveis.^(^ ^14^ ^)^ Alguns autores descreveram a CFPE como sendo uma doença ocupacional, por exemplo, em pacientes expostos a talco^(^ ^15^ ^)^ e em soldadores.^(^ ^16^ ^)^

As vias de sinalização para essas respostas são desconhecidas. Estudos em animais de laboratório demonstraram que o estresse oxidativo que induz a ativação de células inflamatórias, os níveis elevados de metaloproteinases da matriz que causam a atividade proteolítica^(^ ^17^ ^,^ ^18^ ^)^ e a superexpressão de outros mediadores, tais como PDGF,^(^ ^19^ ^)^ TNF-α e TGF-β,^(^ ^20^ ^,^ ^21^ ^)^ são potenciais vias que explicam as lesões que levam a enfisema e fibrose. Um estudo em que se analisaram os mediadores inflamatórios no lavado broncoalveolar de pacientes com FPI mostrou concentrações significativamente maiores das quimiocinas CXCL5 e CXCL8 naqueles que concomitantemente apresentaram enfisema na TCAR.^(^ ^22^ ^)^ Essas quimiocinas estão relacionadas com a acumulação de neutrófilos em espaços aéreos e sugerem uma via diferente de inflamação que leva ao surgimento de alterações enfisematosas sobrepostas à fibrose pulmonar.^(^ ^22^ ^)^

Mutações genéticas têm sido descritas em pacientes com CFPE com e sem carga tabágica significativa.^(^ ^6^ ^,^ ^23^ ^)^ Isso indica que outros fatores de risco além do tabagismo estão relacionados com o surgimento da CFPE ou que o tabagismo pode ser um fator de desencadeamento em pacientes suscetíveis. Vias genéticas plausíveis foram confirmadas em relatos de casos em que foram identificadas mutações no gene da proteína C do surfactante em uma mulher de 32 anos que jamais fumara^(^ ^23^ ^)^ e uma mutação de *ABCA3* em um homem de 41 anos que não era fumante,^(^ ^24^ ^)^ com achados tomográficos idênticos àqueles em pacientes com CFPE. Sabe-se que essas mutações causam disfunção da homeostase do surfactante e, consequentemente, lesão ou morte de células epiteliais alveolares do tipo II e proliferação de miofibroblastos.^(^ ^23^ ^)^ Finalmente, há relatos de características de CFPE em uma família com mutações herdadas da telomerase.^(^ ^25^ ^)^

Esses achados reforçam a ideia de uma combinação de predisposição genética e exposição desencadeante (tabagismo) em indivíduos suscetíveis, levando a dano contínuo a células epiteliais alveolares que não podem ser adequadamente reparadas, iniciando um círculo vicioso de tentativas de regeneração alveolar e ativação descontrolada de proliferação fibrótica e destruição parenquimatosa.^(^ ^26^ ^)^

### Exames de imagem na CFPE

Exames de imagem são essenciais ao diagnóstico da CFPE. Embora a radiografia simples de tórax não seja tão sensível como a TCAR, pode revelar um padrão intersticial predominantemente nas regiões pulmonares subpleurais e basais; a hiperlucência nos ápices pulmonares corresponde a áreas de enfisema. 

A TCAR constitui a base do diagnóstico da CFPE e tipicamente mostra enfisema centrolobular ou parasseptal nos lobos superiores, bem como opacidades reticulares, bronquiectasias de tração, espessamento septal, opacidades em vidro fosco e faveolamento nos lobos inferiores^(^ ^3^ ^)^ (Figuras 1 e 2). Embora a PIU seja o padrão tomográfico mais comum, alguns pacientes apresentam opacidades em vidro fosco que são mais extensas do que o esperado para o padrão de PIU e que, portanto, sugerem a presença de pneumonia intersticial não específica, DPI-BR e mesmo pneumonia intersticial descamativa.^(^ ^3^ ^)^

**Figura 1:** TC de tórax de uma paciente de 67 anos de idade com combinação de fibrose pulmonar e enfisema, mostrando enfisema centrolobular e parasseptal nos lobos superiores (A e B), bem como opacidades em vidro fosco, bronquiectasias de tração e faveolamento nos lobos inferiores (C e D). Nota-se um aspergiloma em uma das bolhas parasseptais no lobo superior direito (seta preta, em B).

**Figura 2:** TC de tórax de um paciente de 70 anos de idade com combinação de fibrose pulmonar e enfisema e exacerbação aguda de doença intersticial. Nota-se enfisema predominantemente parasseptal nos ápices pulmonares, com destruição da arquitetura do parênquima pulmonar (A e B). Extensas áreas de opacidade em vidro fosco e faveolamento podem ser vistas nos lobos inferiores (C e D).

Brillet et al.^(^ ^27^ ^)^ descreveram outros padrões de distribuição de fibrose e enfisema em pacientes com CFPE além daqueles inicialmente descritos: uma transição progressiva de enfisema apical para uma zona de transição entre bolhas e faveolamento; enfisema parasseptal com áreas de fibrose; processos separados com áreas independentes de fibrose e enfisema.^(^ ^27^ ^)^

### Função pulmonar na CFPE

No que tange à função pulmonar de pacientes com CFPE, a espirometria pode ser normal ou mostrar anormalidades leves, com valores de CVF, VEF_1_ e CPT em geral normais ou ligeiramente alterados.^(^ ^3^ ^,^ ^28^ ^)^ A relação VEF_1_/CVF pode estar normal ou ligeiramente abaixo do normal. A DLCO gravemente prejudicada e a hipoxemia durante o exercício são comuns.^(3,28)^

Silva et al. relataram achados semelhantes em uma coorte retrospectiva de 11 pacientes brasileiros, 7 dos quais apresentaram volumes pulmonares normais na espirometria.^(^ ^29^ ^)^ Em nosso ambulatório, foram avaliados 17 pacientes com CFPE, e a espirometria revelou função pulmonar normal e volumes pulmonares normais em 12%, padrão obstrutivo em 18% e padrão restritivo em 47%. Todos os pacientes apresentaram redução da DLCO (Tabela 2).

**Tabela 2:** Resultados dos testes de função pulmonar no momento do diagnóstico em 17 pacientes com combinação de fibrose pulmonar e enfisema tratados no Ambulatório de Doenças Pulmonares Intersticiais do Hospital das Clínicas da Faculdade de Medicina da Universidade de São Paulo entre 2006 e 2013.a

Variável	Resultado
Padrão obstrutivo	3 (18)
Padrão restritivo	8 (47)
Aprisionamento aéreo	4 (24)
Espirometria normal e volumes pulmonares normais	2 (12)
DLCO reduzida	17 (100)
CVF, % do previstob	79 ± 16
VEF1, % do previstob	79 ± 14
VEF1/CVFb	0,77 ± 0,08
CPT, % do previstob	73 ± 17
VR/CPTb	0,36 ± 0,06
DLCO, % do previstob	30 ± 14

a Valores expressos em n (%), exceto onde indicado

b Valores expressos em média ± dp.

Em um estudo de cinco anos de acompanhamento de 16 pacientes com CFPE, o declínio anual da CVF, da DLCO e da relação DLCO/volume alveolar foi significativamente maior do que o encontrado em um grupo de pacientes com DPOC.(30) Em outro estudo, a taxa de declínio do volume pulmonar foi consideravelmente mais baixa em pacientes com CFPE do que naqueles com FPI.^(^ ^31^ ^)^ Embora os valores de DLCO tenham sido menores nos pacientes com CFPE do que nos pacientes com FPI, a taxa anual de declínio da DLCO também foi significativamente menor naqueles. Não houve diferenças entre os dois grupos quanto à sobrevida.^(^ ^31^ ^)^

Uma possível explicação para resultados espirométricos normais ou subnormais não obstante o grave prejuízo da DLCO é que a hiperinsuflação e a maior complacência pulmonar resultantes da perda de elasticidade nas áreas de enfisema podem compensar a perda de volume e complacência pulmonar causada pela fibrose. Outra explicação plausível é que a fibrose impede o fechamento precoce das pequenas vias aéreas observado em pacientes com enfisema.

Embora uma única espirometria possa subestimar a gravidade da doença, Schmidt et al. demonstraram que uma abordagem progressiva, com declínio longitudinal do VEF_1_, pode definir com precisão a progressão da doença e predizer a mortalidade em pacientes com CFPE.^(^ ^32^ ^)^ Além disso, os autores observaram uma correlação entre a extensão do enfisema na TCAR e o declínio do VEF_1_.^(^ ^32^ ^)^

### HAP na CFPE

A prevalência de HAP é extremamente elevada em pacientes com CFPE, e a HAP correlaciona-se com pior sobrevida.^(^ ^3^ ^,^ ^33^ ^,^ ^34^ ^)^ A prevalência de HAP em pacientes com CFPE varia de 47% a 90%, sendo consideravelmente maior do que a observada em pacientes com DPOC ou FPI apenas.^(^ ^18^ ^)^ De fato, a taxa de sobrevida em cinco anos em um estudo com pacientes com CFPE foi de 25% naqueles com HAP (medida por meio de ecocardiografia transtorácica) e de 75% naqueles sem HAP.(28) Em outro estudo, o achado ecocardiográfico de HAP grave relacionou-se com um aumento do risco de morte.^(^ ^35^ ^)^

Mejia et al. realizaram um estudo no México, no qual a HAP foi avaliada por meio de ecocardiografia transtorácica em uma coorte de pacientes com FPI (com e sem enfisema). A HAP não só foi mais prevalente nos pacientes com CFPE como também foi responsável por um prognóstico pior. Outro achado importante foi que a quantidade de enfisema na TC correlacionou-se diretamente com maior pressão sistólica arterial pulmonar.^(^ ^34^ ^)^

Embora a ecocardiografia transtorácica seja uma modalidade de imagem que dependa do operador e que careça de precisão no diagnóstico de HAP em pacientes com doença pulmonar avançada, inclusive DPOC e FPI, parece apresentar boa correlação com medidas de cateterismo cardíaco direito em pacientes com CFPE, sendo, portanto, uma eficaz ferramenta de rastreamento de HAP nesses pacientes.^(^ ^18^ ^,^ ^34^ ^)^

Cottin et al. retrospectivamente caracterizaram a HAP por meio de cateterismo cardíaco direito em 40 pacientes com CFPE. Maior resistência vascular pulmonar, maior FC, menor índice cardíaco e menor DLCO relacionaram-se com um prognóstico pior; a taxa de sobrevida em um ano foi de 60%. Embora a avaliação do efeito do tratamento não tenha sido o objetivo principal do estudo, nenhum dos tratamentos disponíveis melhorou a sobrevida.^(^ ^33^ ^)^

Foram propostos novos métodos não invasivos para o diagnóstico e quantificação de HAP em pacientes com CFPE, incluindo a angiografia por ressonância magnética do tipo *time-resolved*,^(^ ^35^ ^)^ que fornece imagens anatômicas da vasculatura pulmonar e permite a avaliação de parâmetros hemodinâmicos.^(^ ^35^ ^)^ Usando essa técnica, Sergiacomi et al. estudaram prospectivamente 18 pacientes com CFPE usando a média do tempo de trânsito arterial pulmonar e o tempo até o pico de realce como parâmetros substitutos para dados hemodinâmicos (pressão média da artéria pulmonar e resistência vascular pulmonar), obtidos por meio de cateterismo cardíaco direito realizado três dias antes da angiografia por ressonância magnética do tipo *time-resolved*.^(^ ^35^ ^)^ A média do tempo de trânsito arterial pulmonar e o tempo até o pico de realce apresentaram boa correlação com os parâmetros invasivos.^(^ ^35^ ^)^

### Tratamento e prognóstico

Como é o caso da FPI, não há atualmente nenhum tratamento eficaz para a CFPE, exceto pela cessação do tabagismo e o transplante pulmonar (para pacientes com doença avançada). No entanto, broncodilatadores podem ser prescritos para pacientes com resposta positiva a broncodilatadores em testes de função pulmonar. Cottin et al.^(^ ^4^ ^)^ recomendaram o uso de N-acetilcisteína (1,8 g/dia) com base nos resultados de estudos de FPI. Corticosteroides orais e imunossupressores foram considerados uma opção no contexto de CFPE relacionada com DTC; no entanto, nenhum ensaio randomizado foi realizado.^(^ ^4^ ^)^ O transplante pulmonar é a única opção que pode melhorar a sobrevida. 

As principais causas de morte em pacientes com CFPE são a insuficiência respiratória crônica, a HAP,^(^ ^25^ ^,^ ^26^ ^)^ a exacerbação aguda^(^ ^3^ ^,^ ^4^ ^)^ e o câncer de pulmão.^(^ ^36^ ^,^ ^37^ ^)^ Usui et al. encontraram uma prevalência de CFPE de 8,9% em 1.143 pacientes consecutivos com câncer de pulmão primário no Japão.^(^ ^36^ ^)^ Os autores mostraram que, em comparação com pacientes com fibrose ou enfisema apenas, os pacientes com CFPE apresentaram pior sobrevida e maior incidência de exacerbação aguda após cirurgia.^(^ ^36^ ^)^

Variações da DLCO, hipoxemia, baqueteamento digital,(10) pressão média da artéria pulmonar^(^ ^26^ ^,^ ^27^ ^)^ e declínio do VEF_1_^(^ ^32^ ^)^ são considerados melhores substitutos de progressão da doença e maior risco de mortalidade, permitindo uma avaliação mais precoce para o transplante. Recentemente, Chiba et al. demonstraram que dois biomarcadores de fibrose, isto é, KL-6 e proteína D do surfactante, são bons indicadores da extensão da fibrose em pacientes com CFPE.^(^ ^38^ ^)^ Elevados níveis de KL-6 e proteína D do surfactante correlacionaram-se negativamente com todos os volumes pulmonares e com a DLCO.^(^ ^38^ ^)^ Kishaba et al. demonstraram que elevados níveis de KL-6 são preditores de exacerbações agudas em pacientes com CFPE.^(^ ^10^ ^)^

O prognóstico e a mortalidade geral de pacientes com CFPE em comparação com os de pacientes com FPI e DPOC ainda são motivo de debate. Diferentes critérios de inclusão, a duração do acompanhamento, a heterogeneidade dos pacientes (incluindo o tipo de enfisema), a análise retrospectiva dos dados e o viés de tempo ganho (*lead-time bias*) poderiam explicar a heterogeneidade dos resultados. 

Todd et al.^(^ ^39^ ^)^ e Todd & Atamas^(^ ^40^ ^)^ examinaram o grau e o tipo de enfisema em um subgrupo de pacientes com fibrose pulmonar e constataram que os pacientes com uma combinação de fibrose e enfisema centrolobular ou misto (centrolobular e parasseptal) apresentaram melhores taxas de sobrevida do que aqueles com fibrose pulmonar sem enfisema, aqueles com enfisema trivial e aqueles com enfisema parasseptal avançado.^(^ ^39^ ^,^ ^40^ ^)^ O padrão de enfisema e sua extensão parecem correlacionar-se com a gravidade da doença. Os motivos para esses achados permanecem desconhecidos. Uma possível explicação é que os pacientes com CFPE tendem a apresentar volumes pulmonares algo preservados em comparação com os pacientes com fibrose apenas; no entanto, nenhum estudo retrospectivo encontrou qualquer correlação entre volumes pulmonares preservados (determinados por meio de testes de função pulmonar) e melhor sobrevida em pacientes com CFPE. Além disso, não houve diferenças entre os grupos de pacientes supracitados no tocante à DLCO.^(^ ^39^ ^,^ ^40^ ^)^

Outra hipótese para a atuação do enfisema centrolobular como "fator de proteção" baseia-se no fato de que o enfisema centrolobular é essencialmente causado por exposição ao tabaco. Alguns estudos *in vitro* mostraram que as citocinas pró-inflamatórias observadas em casos de tabagismo e enfisema têm propriedades antifibróticas; portanto, tais pacientes poderiam apresentar menores áreas de fibrose e melhor prognóstico.^(^ ^19^ ^)^ O enfisema parasseptal poderia representar outra resposta pulmonar ao tabagismo, levando a fibrose pulmonar grave, ou simplesmente refletir uma maior extensão da fibrose em zonas pulmonares inferiores, exercendo tração no tecido pulmonar localizado nos ápices. No entanto, as áreas de fibrose vistas na radiografia de tórax não precedem o surgimento de enfisema, indicando que ambos os processos provavelmente ocorrem simultaneamente. 

Corroborando essa teoria, Kurashima et al. descobriram que um maior grau de enfisema na TC correlacionou-se com melhores parâmetros de função pulmonar e um melhor prognóstico em comparação com os de um grupo de pacientes com FPI.^(^ ^41^ ^)^ Os autores não classificaram o enfisema em subtipos e sugeriram que o enfisema é um fator protetor em pacientes com CFPE.^(^ ^41^ ^)^ Achados semelhantes foram relatados por Ando et al.^(^ ^42^ ^)^ em um estudo da relação entre a função pulmonar e quantificação tomográfica de enfisema e fibrose na CFPE; os autores concluíram que as alterações fibróticas pulmonares contribuem mais para a progressão da CFPE do que o enfisema.^(^ ^42^ ^)^

As taxas de mortalidade por CFPE e FPI são semelhantes.^(^ ^43^ ^,^ ^44^ ^)^ Recentemente, Ryerson et al. compararam pacientes com FPI-CFPE a pacientes com FPI não CFPE.^(^ ^45^ ^)^ Embora os pacientes com CFPE apresentassem maior carga tabágica, maior necessidade de oxigênio, maior pressão da artéria pulmonar, fisiologia menos restritiva e menor capacidade de difusão pulmonar, não houve diferença significativa entre os dois grupos quanto à mortalidade.^(^ ^45^ ^)^

## Considerações finais

Os pneumologistas estão acostumados a reconhecer a fibrose e o enfisema como duas doenças bem definidas. No entanto, numerosas evidências demonstram que pode haver uma sobreposição; a CFPE é, portanto, uma nova entidade, com características únicas. Em vista do fato de que a maioria dos estudos que investigaram a CFPE foi retrospectiva, são necessários mais estudos sobre o papel do enfisema e seus subtipos, a progressão da fibrose/enfisema e sua correlação com a inflamação, as opções de tratamento (incluindo o tratamento da HAP) e o prognóstico em pacientes com CFPE. Necessita-se urgentemente de maior compreensão da fisiopatologia e progressão da CFPE a fim de melhorar seu tratamento, já que atualmente não há nenhum tratamento eficaz para a doença.
